# The angiotensin-converting enzyme (ACE) gene family of *Anopheles gambiae*

**DOI:** 10.1186/1471-2164-6-172

**Published:** 2005-12-05

**Authors:** Susan Burnham, Judith A Smith, Alison J Lee, R Elwyn Isaac, Alan D Shirras

**Affiliations:** 1 Faculty of Biological Sciences, Miall Building, University of Leeds, Clarendon Way, Leeds LS2 9JT, UK; 2 Department of Biological Sciences, Lancaster Environment Centre, Lancaster University, Lancaster, LA1 4YQ, UK

## Abstract

**Background:**

Members of the M2 family of peptidases, related to mammalian angiotensin converting enzyme (ACE), play important roles in regulating a number of physiological processes. As more invertebrate genomes are sequenced, there is increasing evidence of a variety of M2 peptidase genes, even within a single species. The function of these ACE-like proteins is largely unknown. Sequencing of the *A. gambiae *genome has revealed a number of ACE-like genes but probable errors in the Ensembl annotation have left the number of ACE-like genes, and their structure, unclear.

**Results:**

TBLASTN and sequence analysis of cDNAs revealed that the *A. gambiae *genome contains nine genes (*AnoACE *genes) which code for proteins with similarity to mammalian ACE. Eight of these genes code for putative single domain enzymes similar to other insect ACEs described so far. AnoACE9, however, has several features in common with mammalian somatic ACE such as a two domain structure and a hydrophobic C terminus. Four of the *AnoACE *genes (2, 3, 7 and 9) were shown to be expressed at a variety of developmental stages. Expression of *AnoACE3*, *AnoACE7 *and *AnoACE9 *is induced by a blood meal, with *AnoACE7 *showing the largest (approximately 10-fold) induction.

**Conclusion:**

Genes coding for two-domain ACEs have arisen several times during the course of evolution suggesting a common selective advantage to having an ACE with two active-sites in tandem in a single protein. AnoACE7 belongs to a sub-group of insect ACEs which are likely to be membrane-bound and which have an unusual, conserved gene structure.

## Background

In mammals, angiotensin-converting enzyme (EC 3.4.15.1, ACE, peptidyl-dipeptidase A), an important member of the M2 peptidase family, is found on the surface of endothelial cells and is best known for its role in the biosynthesis of angiotensin II, as well as the degradation of circulating bradykinin and the haemoregulatory peptide, N-acetyl SDKP [1-3]. Mammalian ACE exists as two isoforms, a somatic form (sACE, 150–180 kDa) and a smaller protein (germinal ACE, 90–110 kDa) found exclusively in adult testes. The ACE gene comprises twenty-six exons and two promoters, and has clearly arisen by gene duplication [4]. The somatic promoter drives expression of the larger protein (exons 1–12 and exons 14–26), which consists of two very similar domains connected in tandem by a short inter-domain peptide. Each domain possesses a functional peptidase active site and they are commonly called the N- and C-domains after their relative position to the amino and carboxy termini. The bulk of the protein including the amino terminus is extracellular and is linked by a hydrophobic transmembrane sequence to a short intracellular peptide. The biological rationale for two catalytic units linked in tandem is not known, although there is experimental evidence to suggest that co-operativity exists between the two halves of the protein [5-7].

In developing spermatids, an alternative intragenic promoter utilises exons 13–26 and generates a single domain ACE (gACE) which is largely identical to the C-terminal domain of somatic ACE [4,8]. Male mice lacking gACE are infertile as a result of sperm that are defective in migration in the oviduct and in binding to the zona pellucida [9].

Insect ACE has broad substrate specificity and its presence in the haemolymph of insects raises the possibility that, like mammalian sACE, it is required for extracellular metabolism of peptide hormones [10,11]. *Drosophila melanogaster *has six ACE-like genes (*Ance, Acer*, *Ance-2, Ance-3, Ance-4 *and *Ance-5*), all coding for single domain proteins. The ANCE protein has been relatively well characterised and has been shown to have several biochemical similarities to the mammalian enzyme. Its activity is developmentally regulated, with highest levels during metamorphosis [12,13]. ANCE is also expressed in spermatocytes and appears to have an important role in spermiogenesis [14]. Of the other *Drosophila *ACEs, only the *Acer *gene product has been studied biochemically and it is likely that ANCE-2, ANCE-3, ANCE-4 and ANCE-5 do not function as peptidases since they lack one or more of the residues that are essential for peptidase activity [15]. ACER, like ANCE and human ACE, is a peptidyl dipeptidase, but is generally less efficient than ANCE at cleaving dipeptides from many oligopeptide substrates [16]. *Acer *is expressed in the embryonic heart [17] and in both the male and female gonads and brain of adult flies (A. Carhan, R. E. Isaac and A. D. Shirras, unpublished results) where it is assumed to have a role in the metabolism of, as yet unidentified, biologically active peptides involved in neuroendocrine signalling and reproduction.

ACE activity has been found in the gonads and accessory glands of several insect species in addition to *D. melanogaster*, suggesting a conserved role for this enzyme in insect reproduction [10,11]. Recently, we have provided evidence for a role for ACE in reproduction in the mosquito, *A. stephensi*. ACE activity in the adult female mosquito increases by 260% following a blood meal [10,18] and it has been proposed that the induced ACE has a role in regulating peptide signalling in response to a blood meal. We have shown that feeding two different ACE inhibitors to male *A. stephensi *in their glucose diet, resulted in approx. 80% reduction in the number of eggs laid [10]. In addition, ACE inhibitors introduced in the blood meal, resulted in a dose-dependent effect on brood-size, but had no effect on oocyte development, nor the rate of digestion of the blood. The only observable difference between inhibitor-fed and control insects was that the inhibitor-fed females could not lay eggs, even 8 days after the blood meal, suggesting that the blood-induced ACE is involved in the control of egg-laying [19].

To understand the mechanism of ACE induction in blood-fed mosquitoes and the role of the enzyme in mosquito reproductive physiology, we have characterised the ACE gene family in *Anopheles gambiae*, a mosquito responsible for the transmission of the human malaria parasite and whose genome has recently been sequenced [20]. The partially annotated *A gambiae *genome [21] reveals between eight and twelve potential ACE-like genes. We now report the correct number of *A. gambiae *ACE (*AnoACE*) genes, confirm their corresponding protein sequences and establish their expression patterns during development and in response to a blood meal. We show that several of the AnoACEs are similar to other insect ACEs, but that AnoACE9 is a novel two-domain ACE whose structure resembles that of human somatic ACE.

## Results

### ACE-like genes in the *A. gambiae *genome

**Figure 1:**
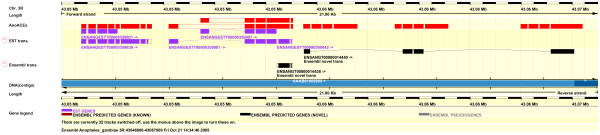
**Annotation of ACE-like genes in the 35C cluster**. Ensembl ContigView display of the 43.05–43.07 Mb region of chromosome 3R showing AnoACE custom track annotation (red bars), Ensembl EST transcripts (ENSANGESTT) and Ensembl predicted transcripts (ENSANGT). From left to right the *AnoACE *genes are numbered *2*, *3 *(with two alternative 5' exons), *4*, *5 *and *6*.

The Ensembl annotation of the *A. gambiae *genome (version 34.2 g) [21] suggests that there are eight genes encoding proteins belonging to the M2 (ACE) family of peptidases. BLAST searching of the genome and an examination of the predicted genes, however, shows that not all ACE-like proteins have been predicted, while several of those that have been identified have missing exons, or are hybrids, composed of exons from more than one ACE-like gene. These problems are particularly acute for a cluster of ACE-like sequences at 35C on chromosome 3R (Fig. 1). In order to confirm the number and organisation of ACE-like genes in the *A. gambiae *genome a combination of TBLASTN analysis [21], using *Drosophila melanogaster *ACE proteins as query sequences, and cDNA sequence analysis was carried out. Results are summarised in Tables 2 and 3 and may be viewed as a custom track in Ensembl Mosquito [37].

**Table 2:** ACE-like genes in the *A. gambiae *genome. Genes were identified by a combination of TBLASTN using *Drosophila *genes as query sequences and cDNA sequence analysis. Corresponding Ensembl (version 34.2 g) transcripts and ESTs (where known) are indicated.

*AnoACE*	ENSANGT	ENSANGESTT	Chromosome	Contig	Coordinates
1	0000010993	-	2L, 28C	AAAB01008807_41	47,788,338–47,790,069
2	-	00000359639 00000359801	3R, 35C	AAAB01008980_249	43,046,775 – 43,049,518
3	00000014438 (part)	0000035981* 00000359997* 00000360042	3R, 35C	AAAB01008980_249	43,050,199 – 43,055,041
4	00000014440 (part)	-	3R, 35C	AAAB01008980_249	43,055,473 – 43,057,570
5	00000014440 (part)	-	3R, 35C	AAAB01008980_249	43,059,057 – 43,061,130
6	00000014440 (part)	-	3R, 35C	AAAB01008980_249	43,063,988 – 43,066,391
7	00000022325 00000014398^†^ (part)	00000360081	3R, 35C	AAAB01008980_250 AAAB01008980_252^†^	43,068,658 – 43,072,250 43,136,048 – 43,139,362^†^
8	00000007115	-	3R, 29C	AAAB01008964_349	3,659,796 – 3,661,800
9	00000028617 00000011721	00000365570 00000365630	2R, 19C	AAAB01008898_151	59,413,237 – 59,418,650

*alternative transcripts with different 5' exons

† 3' exons of *AnoACE7*, 69 kb downstream of the rest of the gene

**Table 3:** Exon coordinates of *AnoACE *genes. Coordinates are as in Ensembl Mosquito version 34.2 g. Exon annotations may be viewed as a custom track in Ensembl Mosquito [37].

*AnoACE*, Chr.	Exon	Start	End	Length	Start codon	Stop codon
1, 2L	1	47788338	47790069	1731	47788338	47790069
2, 3R	1	43046775	43046948	173	43047058	43049381
	2	43047050	43047327	277		
	3	43047407	43047771	364		
	4	43047832	43048053	221		
	5	43048118	43048261	143		
	6	43048324	43049032	708		
	7	43049101	43049274	173		
	8	43049343	43049518	175		
3A, 3R	1	43050199	43050555	356	43050288	43054998
	2	43053178	43053542	364		
	3	43053612	43053976	364		
	4	43054035	43054916	881		
	5	43054981	43055041	60		
3B, 3R	1	43051447	43051757	310	43051523	43054998
	2	43053178	43053542	364		
	3	43053612	43053976	364		
	4	43054035	43054916	881		
	5	43054981	43055041	60		
4, 3R	1	43055473	43055722	249	43055473	43057570
	2	43055795	43056159	364		
	3	43056227	43056590	363		
	4	43056672	43057570	898		
5, 3R	1	43059057	43059300	243	43059057	43061130
	2	43059368	43059732	364		
	3	43059801	43060165	364		
	4	43060238	43061130	892		
6, 3R	1	43063988	43064231	243	43063988	43066391
	2	43064299	43064663	364		
	3	43064732	43065096	364		
	4	43065169	43066391	1222		
7, 3R	1	43068658	43069121	463	43068773	43139362
	2	43069190	43069805	615		
	3	43070472	43070631	159		
	4	43070704	43070827	123		
	5	43070892	43071025	133		
	6	43071102	43071687	585		
	7	43071770	43072014	244		
	8	43072151	43072250	99		
	9	43136048	43136189	141		
	10	43139040	43139362	322		
8, 3R	1	3659796	3661191	1395	3659796*	3661800
	2	3661258	3661542	284		
	3	3661607	3661800	193		
9, 2R	1	59413237	59413683	446	59413677	59418197
	2	59414399	59414615	216		
	3	59414676	59416593	1917		
	4	59416658	59418650	1992		

*Start of open reading frame, no start methionine identified

**Figure 2:**
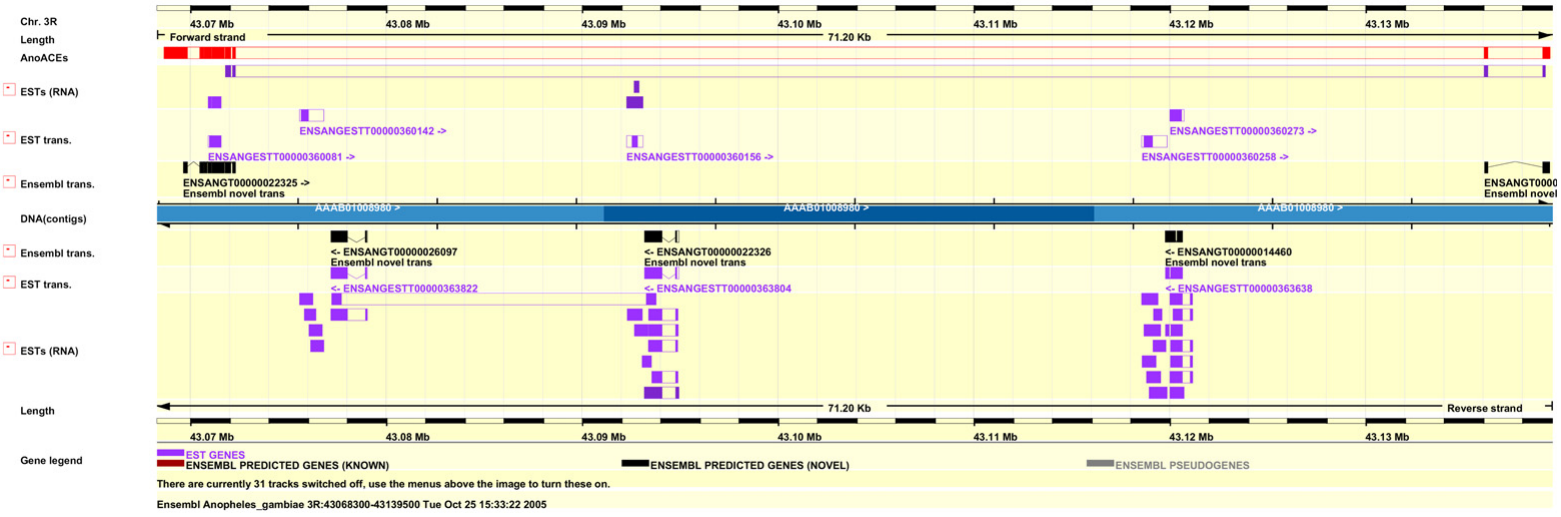
**Annotation of *AnoACE7***. Ensembl ContigView display of the 43.07–43.13 Mb region of chromosome 3R showing AnoACE custom track annotation (red bar), Ensembl ESTs (purple bars), EST transcripts (ENSANGESTT) and Ensembl predicted transcripts (ENSANGT). Eight upstream exons, encoding the bulk of the protein, are situated at the 3' end of the 35C cluster, downstream of *AnoACE6 *(Fig. 1). Two exons encoding the C terminus of the protein are situated 69 kb downstream. The sequence of EST clone P141-G-08-5 spans upstream and downstream exons of *AnoACE7 *and is shown below the gene annotation. The large intron contains several potential genes. Not all introns are shown at this scale.

TBLASTN analysis revealed 11 possible ACE-like coding sequences. Six of these sequences are arranged in a tightly linked tandem cluster at 35C (Figs. 1 &2). These were numbered (from 5' to 3') *AnoACE 2,3,4,5,6 *and *7*. Several EST clones exist for *AnoACE2 *and *AnoACE3*, allowing the exons of these genes, and their coding regions to be unambiguously assigned. *AnoACE3 *has two alternative transcripts which differ in their first exon. No cDNA clones are available for *AnoACE*s *4*, *5 *and *6*. The exons of these genes were identified by BLAST similarity and the SNAP exon predictions of Ensembl [21]. The nucleotide sequences of *AnoACE*s *5 *and *6 *are 97% identical, including the introns, which raised the possibility that these two genes are the result of a sequence assembly error caused by nucleotide polymorphisms in the PEST strain. This high level of identity is only observed within the putative transcribed region, however. The upstream flanking sequence of *AnoACE6 *shares only around 75% identity with the *AnoACE5 *upstream DNA and there is no significant similarity between the downstream flanking sequences of the two genes. An assembly error can therefore be ruled out.

The *AnoACE7 *gene has an unusual organisation; there are eight upstream exons situated at the 3' end of the 35C cluster which encode the bulk of the protein and there are two exons encoding the C terminus of the protein 69 kb downstream (Fig. 2). These exons were shown to belong to the *AnoACE7 *gene by the sequence of NAP1 cDNA P141-G-08-5 which spans exon 8 and the two downstream exons (Fig. 2., Ensembl annotation). The gene structure was further confirmed by TBLASTN alignment with the *Drosophila *ANCE-3 protein. The 5' end of *AnoACE7 *was determined by RT-PCR analysis using nested primers designed against the genomic sequence. A 1.1 kb RT-PCR product was cloned, sequenced and found to contain a long open reading frame coding for a protein which overlapped existing predicted AnoACE7 protein sequence and which had a putative signal peptide. This was assumed to correspond to the N terminus of the AnoACE7 precursor.

**Figure 3:**
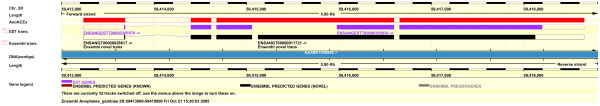
**Annotation of *AnoACE9***. Ensembl ContigView display of the 59.413–59.419 Mb region of chromosome 2R showing AnoACE custom track annotation (red bar), Ensembl EST transcripts (ENSANGESTT) and Ensembl predicted transcripts (ENSANGT). The *AnoACE9 *gene consists of 4 exons. Exons 3 and 4 both contain ACE active site coding regions and are spanned by cDNA 19600449703686 (ENSANGESTT00000365630) indicating a double-domain enzyme.

The *AnoACE9 *gene contains two ACE-like coding regions. The sequence of cDNA 19600449703686 (MRA-468-36) spans both coding regions (Ensembl annotation), indicating that this is a single gene coding for a double-domain protein, rather than two separate genes (Fig. 3).

### AnoACE proteins

**Figure 4:**
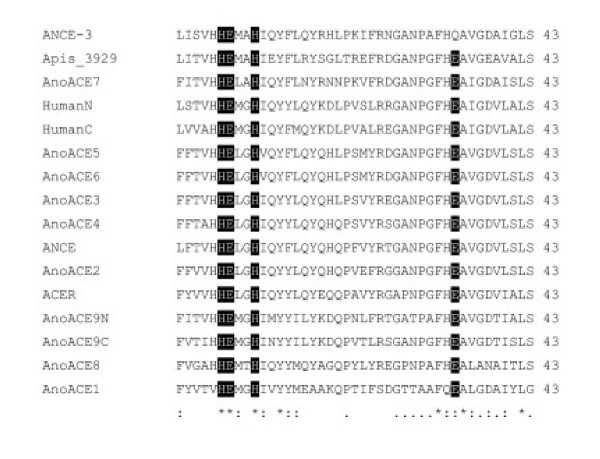
**Active site region of ACE-like proteins**. The active site regions of AnoACEs, the N and C domains of human ACE, *D. melanogaster *ANCE, ACER and ANCE-3, and *Apis *3929 were aligned using CLUSTALX [36]. Amino acids shown to be required for activity in mammalian ACE are highlighted. The histidines in the HExxH motif are required for zinc coordination, whereas the glutamate is required for catalysis. The downstream glutamate is required for zinc coordination. This is replaced by glutamine in ANCE-3.

Protein sequences were predicted from cDNAs for AnoACEs 2, 3, 7 and 9. AnoACEs 1, 4, 5, 6 and 8 were predicted from genomic sequence and alignment with *Drosophila *ACE proteins. Predicted properties of the proteins are summarised in Table 4. Signal peptides were identified for all proteins except AnoACEs 1 and 8. All of the *AnoACE *genes, except for *AnoACE9*, code for single domain proteins similar to those previously found in insects. AnoACE9 is a putative double domain enzyme, similar to mammalian somatic ACE. Two of the AnoAces (7 and 9) have hydrophobic C termini which might serve as membrane anchors or be lost as a result of post-translational modification with a GPI anchor [22,23]. AnoACE7 has an extended N terminus, compared to the other proteins. All proteins have a full complement of required active site amino acids in the HExxH...EAV/I/L motif (Fig. 4). Predicted protein sequences are available as additional data file 1.

**Table 4:** Predicted AnoACE proteins. Conceptual proteins were translated from cDNA sequences for AnoACEs 2, 3, 7 and 9 and from genomic sequence for AnoACEs 1, 4, 5, 6, and 8.

AnoACE	Length (aa)	Active sites	Hydrophobic C terminus	Predicted GPI anchor
1^‡^	576	1	No	No
2	638	1	No	No
3A*	630	1	No	No
3B*	619	1	No	No
4	625	1	No	No
5^†^	619	1	No	No
6^†^	730	1	No	No
7	917	1	Yes	No/marginal
8^‡^	623	1	No	No
9	1225	2	Yes	Yes

*AnoACE3A and AnoACE3B are derived from alternative transcripts and differ at their N termini

†AnoACE5 and AnoACE6 are 97% identical, except for an extended C terminus in AnoACE6

‡N-terminus probably missing, no signal peptide

### Comparative genomics of ACE-like genes

**Figure 5:**
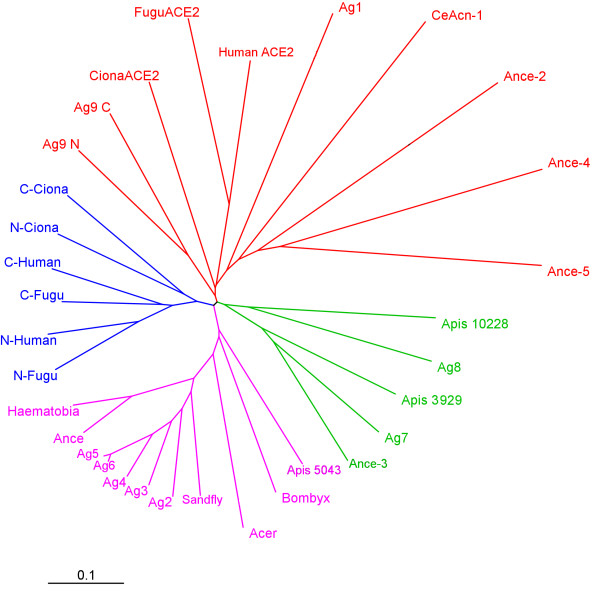
**Evolutionary relationships of invertebrate and chordate ACE-like proteins**. Core sequences (corresponding to amino acids161 to 569 of the AnoACE2 pre-protein) of each protein were aligned and a neighbour-joining tree calculated using CLUSTALX [36]. TreeView was used to produce a radial tree. Sequences used were AnoACEs (Ag)1, 2, 3, 4, 5, 6, 7, 8, and 9 (N and C domains); *D. melanogaster *ANCE, ACER, ANCE-2, -3, -4 and -5; *B. mori *ACE (accession no. BAA97657); *H. irritans *ACE (accession no. Q10715); *C. elegans *ACN-1; N and C domains of Human ACE, *Fugu rubripes *(Ensembl SINFRUP00000174092) ACE; *A. mellifera *Ensembl proteins ENSAPMP00000005043, ENSAPMP00000003929 and ENSAPMP000000010228; Sandfly (*Lutzomyia longipalpis*) ACE (accession no. AAS16911); Human and *F. rubripes *(Ensembl SINFRUP00000161972) ACE2. N and C domains of *Ciona intestinalis *ACE, were assembled from genomic sequence in contig AABS01000302.1. Scale bar shows number of amino acid substitutions per site.

A phylogram showing the relationships of the AnoACEs to other insect and chordate ACEs is shown in Fig. 5. AnoACEs 2, 3, 4, 5 and 6 cluster with *Drosophila *ANCE and ACER, and ACE-like proteins from *Apis mellifera*, *Haematobia irritans*, *Lutzomyia longipalpis *and *Bombyx mori*. These proteins are all single domain proteins which lack a hydrophobic C terminus. A second cluster of insect proteins contains AnoACEs 7 and 8, the two remaining *Apis *ACEs and *Drosophila *ANCE-3. AnoACE7 is a one-to-one orthologue of ANCE-3 and of *Apis *3929. AnoACE8 is a one-to-one orthologue of *Apis *10228. There appears to be no *Drosophila *orthologue of AnoACE8. The similarity of the AnoACE7 group extends to the genomic organisation of the genes. In each case the bulk of the protein is encoded by a set of upstream exons and these are separated from the downstream exons, encoding the C terminus of the protein by a large intron. In the case of *Anopheles *the intron is 69 kb in length, in *Drosophila *35 kb and in *Apis *21 kb. The proteins in this group are characterised by an extended N terminus, rich in proline and charged amino acids. AnoACE7 and *Drosophila *ANCE-3 have a hydrophobic C terminus but this is absent from the predicted *Apis *protein. GPI anchor prediction software [23] predicts no GPI anchor for AnoACE7 whereas *Drosophila *ANCE-3 has a strong GPI anchor prediction.

**Figure 6:**
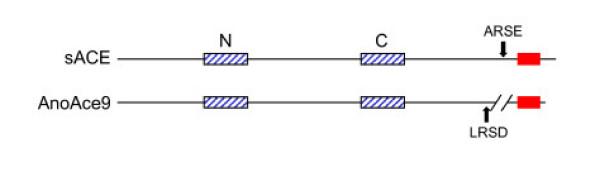
**Diagrammatic alignment of human somatic ACE (1306 amino acids) and AnoACE9 (1225 amino acids)**. The hatched boxes represent the N and C domain active site regions. Filled boxes are the C-terminal hydrophobic potential membrane anchor regions. AnoACE9 has a very short potential cytoplasmic region and a sequence gap relative to the human enzyme on the N terminal side of the putative transmembrane region. Arrows indicate potential secretase recognition sequences.

AnoACEs 1 and 9 are placed in a cluster with a diverse group of ACE-like proteins that includes *Drosophila *ANCEs-2, -4 and -5, the *C. elegans *ACE-like protein ACN-1 and vertebrate ACE2. The latter protein, while having sequence similarity to ACEs, has a different enzymatic activity and acts as a carboxypeptidase rather than a dipeptidase. Several members of this group (*Drosophila *ANCEs-2, -4 and -5, ACN-1) lack one or more essential active site amino acids and are therefore unlikely to be active peptidases. AnoACE9 has several structural similarities with chordate ACEs. It has two active site domains and a hydrophobic C terminus with a strong GPI anchor prediction (Fig. 6). The N and C domains of this protein are more similar to each other than to other ACEs, unlike the vertebrate N and C domains which cluster together.

### Expression of AnoACE genes

**Figure 7:**
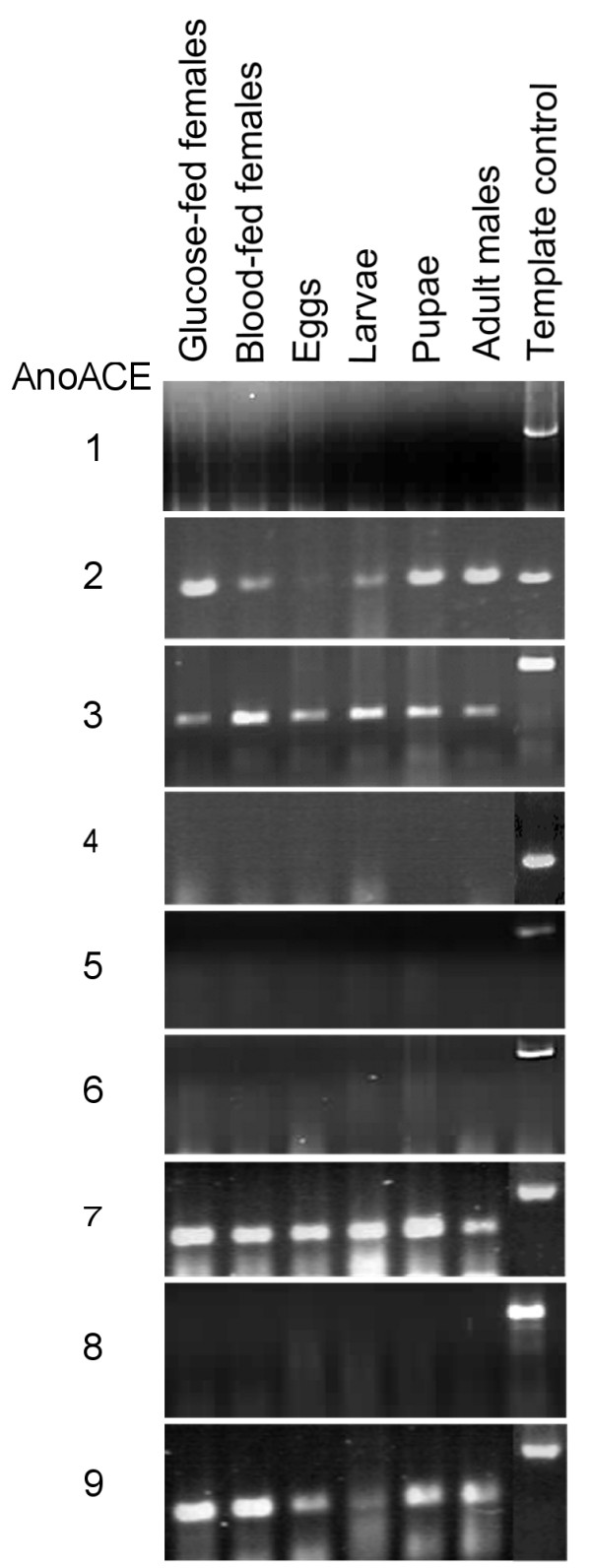
**RT-PCR analysis of *AnoACE *gene expression**. RNA was extracted from glucose fed adult females, blood fed adult females, eggs, larvae, pupae and adult males. Control templates for amplification were genomic DNA except for AnoACE2 primers when the template was DNA from cDNA clone 19600449622752 (MRA-467-39). Amplification products were observed for *AnoACE*s *2*, *3*, *7 *and *9 *only.

Primers for all *AnoACE *genes were validated by PCR amplification of genomic DNA or cDNA. Each primer pair produced a product of the expected size (Fig. 7).

For semi-quantitative RT-PCR analysis RNA was extracted and cDNA produced from different developmental stages of mosquitoes, namely, eggs, larvae, pupae, adult males, glucose-fed females and blood-fed females. Figure 7 shows that *AnoACE*s *2*, *3*, *7 *and *9 *are expressed at all developmental stages tested, whereas no PCR product was observed for *AnoACE*s *1*, *4*, *5*, *6 *and *8*. The authenticity of the products from *AnoACE2*, *3*, *7 *and *9 *was confirmed by cloning into the pGEM T-Easy^® ^vector and sequencing.

**Figure 8:**
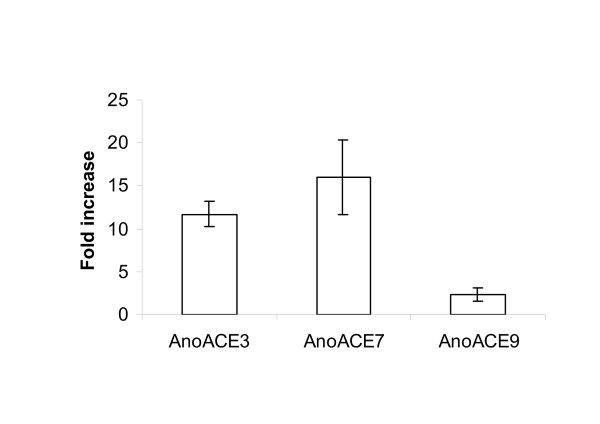
**Relative expression of *AnoACE *genes in glucose and blood-fed females**. RNA was extracted from three different cohorts of mosquitoes, either after continuous glucose feeding or 48 hours after a blood meal, and analysed by quantitative RT-PCR. The mean increase in gene expression was 11.6 fold for *AnoACE3*, 16 fold for *AnoACE7 *and 2.4 fold for *AnoACE9*.

Gene expression for all the AnoACE genes was analysed in glucose and blood-fed females using quantitative real-time RT-PCR. Other than *AnoACE*s 2,*3*,*7 *and *9*, expression levels were as for no template controls. Transcription of *AnoACE3*, *AnoACE7 *and *AnoACE9 *was shown to be up-regulated 48 hours following a blood meal (Fig. 8). The increase in transcription was consistent for three separate cohorts of female mosquitoes.

## Discussion

Our analysis of the *A. gambiae *genome has revealed nine ACE-like genes. This is the largest number of ACE genes for any species with a complete genome sequence. *Drosophila melanogaster *has six ACE-like genes [15], but only two of these (*Ance *and *Acer*) are known to code for active peptidase proteins. As well as having the ACE-like genes found in *D. melanogaster*, *D. pseudoobscura *has an additional *Ance*-like gene which is likely to code for an active peptidase, since all the amino acids that are necessary for catalysis and coordination with the active site zinc are conserved (A.D. Shirras, unpublished work). *Apis mellifera *has three ACE-like genes, each with a full complement of the critical active site amino acids [24]. At present we have evidence that at least four *AnoACE *genes (*2*, *3*, *7 *and *9*) are expressed, but have no evidence for expression of *AnoACE*s *1*, *4*, *5*, *6 *and *8*. All of the *AnoACE *genes, however, have complete open reading frames and the corresponding proteins have a full complement of essential active site amino acids, so we cannot rule out the expression of the other genes at low levels, in a small number of cells or at stages that we have not examined.

Some of the *AnoACE *genes have clearly arisen by recent gene duplications. The cluster at 35C contains five genes that are closely related in sequence. Two of these (*AnoACE5 *and *AnoACE6*) are almost identical. Similar clusters of other duplicated genes exist within the *A. gambiae *genome; for example there is a cluster of M14 carboxypeptidase genes at 29D which contains adjacent genes with greater than 90% identity. In both these clusters the high levels of identity are restricted to the transcribed regions with flanking DNA showing lower levels of similarity. This suggests that selective pressures are maintaining the nucleotide sequences of these genes and that they are expressed at some stage.

Comparison of ACE protein sequences (Fig. 5) suggests that there are three functional groupings in insects. The first contains *Drosophila *ANCE, AnoACEs 2, 3, 4, 5 and 6 and several other insect ACEs. These are single domain, secreted enzymes. This class of ACE proteins is similar to the chordate N and C domains, both in terms of sequence and enzyme activity. The second group includes AnoACE7, *Drosophila *ANCE-3 and *Apis *3929. These proteins are single domain, but have extended N termini. The *Drosophila *and mosquito proteins have a hydrophobic C terminus suggesting that they may be membrane-tethered. The active sites of these three proteins are characterised by an alanine in place of glycine at position 4 of the HExxH motif (Fig. 4). AnoACE8 and *Apis *10228 form diverged members of this group.

The third group of insect ACEs, containing AnoACE1 and *Drosophila *ANCE-2, -4 and -5, is highly divergent. These appear to be secreted proteins and, whereas AnoACE1 may be enzymically active, the *Drosophila *examples lack essential active site amino acids. *C. elegans *ACN-1 and vertebrate ACE2 proteins form highly diverged members of this family. This result is to be expected as they have distinctive and highly evolved roles in these animals [25,11].

AnoACE9 is highly unusual. Phylogenetic analyses using several different tree construction methods (Neighbour-Joining, Maximum Parsimony, Minimum Evolution, Unweighted Pair Group Method with Arithmetic Mean) do not place it convincingly in any of the insect groups described above. In terms of overall structure it is more similar to mammalian somatic ACE than to any invertebrate ACE described so far (Fig. 6). It has two active site domains and has a hydrophobic C terminus. This similarity with vertebrate ACEs is, as far as we know, unique amongst non-chordate invertebrates. It is likely that AnoACE9 is membrane bound, either by virtue of its hydrophobic C-terminus or via a potential GPI anchor. In mammals, membrane sACE can be released from the plasma membrane by unidentified metalloproteases, known as secretases or sheddases, that cleave the Arg-Ser bond in the Ala-Arg-Ser-Glu motif of the juxtamembrane stalk [26,27]. Interestingly, a similar peptide sequence (Leu-Arg-Ser-Asp) is found in the same position in the proposed juxtamembrane stalk of AnoACE9 (Fig. 6), which might serve as a cleavage site for a mosquito ACE secretase.

In mammals, the two active sites of sACE are involved in the conversion of angiotensin I to angiotensin II and the hydrolysis of bradykinin, but only the N-domain is involved in N-acetyl SDKP metabolism [2]. Thus, duplication appears to have provided an opportunity for the N-domain to acquire distinctive substrate specificity. However, this does not explain why mammalian sACE is a single protein with two catalytic domains joined by a peptide linker, rather than single genes encoding single domain proteins as occurs in *D. melanogaster*. Our phylogenetic analysis indicates that the gene duplication event leading to the two-domain vertebrate somatic ACE, occurred around 450 million years ago, before the divergence of amphibians and fishes, and that two other very similar events occurred independently to give rise to a two-domain enzyme: one in the lineage leading to *A. gambiae *and one in the urochordate lineage leading to *Ciona intestinalis *(Fig. 5). That is, two-domain ACE proteins have been selected for on three separate occasions, suggesting that there is a distinct functional advantage to having two active sites organised in this manner over two single-domain proteins. Support for this hypothesis comes from several studies on mammalian sACE that suggest that the two active sites can have cooperative effects on ACE activity [5-7]. Recent studies indicate that the N-domain of human somatic ACE also has a negative effect on the shedding of the protein from the cell surface by cleavage within the stalk region that separates the C-domain from the membrane anchor [28]. More detailed biochemical studies on AnoACE9 are now required to determine whether similar cooperative phenomena occur in mosquitoes.

The expression of *AnoACE*s *3*, *7 *and *9 *is induced by a blood meal. Two of these genes (*AnoACE 3 *and *7*) are induced more than ten-fold. Neuropeptides are known to be important for mediating several behavioural and physiological responses to a blood meal in mosquitoes [29-34]. Our previous work showing that ACE inhibitors block egg-laying, but not egg development, in blood fed *A. stephensi *[19] led us to propose that the elevated peptidase activity might be important for regulating a peptide signal involved in egg-laying. The up-regulation of *AnoACE*s *3*, *7 *and *9 *following a blood meal in *A. gambiae*, raises the possibility that ACE may also be involved in regulating post feeding oviposition in this species.

A role for at least some AnoACEs in the immune response is suggested by recent microarray data [35]. *AnoACE7 *(ENSANGT00000022325) was shown to be 2.4-fold upregulated by challenge with *Salmonella typhimurium*. *AnoACE1 *(ENSANGT0000010993) was 3.5-fold upregulated by *Staphylococcus aureus *infection, whereas the expression of *AnoACE9 *(ENSANGT00000028617) was reduced 2.4-fold by *Beauveria bassiana*. We were unable to detect *AnoACE1 *expression in a variety of developmental stages. However, the expression of this gene suggested by the infection microarray may mean that this gene plays a specific role as a component of the immune response.

## Conclusion

The *Anopheles gambiae *genome contains 9 genes coding for proteins with similarity to mammalian ACE. Six of these genes are found in a tandem cluster situated at 35C on chromosome 3R. Five of the members of this cluster code for proteins which are similar to previously described insect ACEs: they contain a single active site domain and lack a C-terminal hydrophobic region. *AnoACE7*, situated at the 3' end of the 35C cluster, is an orthologue of *Drosophila Ance-3 *and of *Apis *gene ENSAPMG00000014390. In all three species, there is a similar gene structure with the bulk of the coding exons separated from the C-terminal exons by a large intron which contains other genes. The AnoACE7 protein, like *Drosophila *ANCE-3, has a hydrophobic C-terminus and is likely to be membrane bound. AnoACEs 8 and 10 are also predicted to be single domain proteins but AnoACE9 is unique amongst insect ACEs described so far in that it contains two active site domains and has a hydrophobic C-terminus, a structure similar to that of mammalian somatic ACE. Expression of only 4 of the *Anopheles *ACE genes (2, 3, 7 and 9) could be detected by RT-PCR in the stages tested. Expression of AnoACEs 3, 7 and 9 increased following a blood meal.

## Methods

### Insects

Male and female *Anopheles gambiae *were maintained at a temperature of 26°C and at a relative humidity of 80 %. Newly hatched adult mosquitoes were fed for seven days on sterile 5% (w/v) glucose/0.5% (w/v) p-aminobenzoic acid solution [20]. On the seventh day female mosquitoes were harvested and divided into two cohorts. Mosquitoes in cohort A were maintained on the glucose/p-aminobenzoic acid solution, whilst mosquitoes in cohort B were fed mouse blood. After the blood meal any unfed female mosquitoes (from cohort B) were discarded. Mosquitoes from cohort B were collected 48 hours after the blood meal. Insects from cohort A were collected 48 hours after cohort B was given the blood meal.

### Extraction of nucleic acids

Nucleic acids were extracted using either the Qiagen DNeasy^® ^or RNeasy^® ^kits as per manufacturer's directions. During RNA extraction the optional RNase-free DNase I from Qiagen^® ^was used to digest DNA. After quantification, extracted DNA was stored at 4°C and RNA at -80°C until required.

### PCR

Primers were designed for each possible mosquito ACE gene, with each pair crossing exon boundaries, where this was possible (see Table 1 for primer sequences).

**Table 1:** Primers for PCR of *AnoACE *genes

Gene	Forward Primer sequence (5'-3')	Reverse Primer Sequence (5'-3')	Predicted Product Size From Genomic DNA(bp)
*AnoACE1*	GGAATGCATCGTGTGTGTTC	TACTCGAAGGGAATGGTTGG	943
*AnoACE2*	GGTACCGATCAACCAGTGCT	AAATCGCATCGACAAGCTCT	440
*AnoACE3*	CCCTGTGCAAGTGCTGATAA	ATACGGAACGCCATTGGATA	214
*AnoACE4*	CAGCCGAGATGCTGAAGAGT	GAACTCGCGCAACCTATACC	328
*AnoACE5*	TTGCTGCAGCGAATGCTAT	CACACCAGCCCAATCTTGT	754
*AnoACE6*	CAGCGAATGCTGTGTTCCT	CAATGTTGCCCCACTTCTGT	748
*AnoACE7*	CGCAGTGGAACTTTGAGACA	GTCTATTCGGCGCATTTGAT	782
*AnoACE8*	GGTCTGGGCGATACCAAGTA	CGCGTCAAATCGGAGTTAAT	220
*AnoACE9*	GCACACGTATCGGAAAATGA	GCGCTTCTCGATGAACTACC	803

The primers were also designed so that the product size from cDNA would be approximately 100 bp as appropriate for use with QPCR, except for *AnoACE1 *where the product was 943 bp. Amplification reactions were prepared in 20 μl volumes and contained; 100 ng of template, 2 μl of PCR buffer (670 mM Tris-HCl, pH 8.8; 166 mM [NH_4_]_2_SO_4_; 67 mM MgCl_2_), 200 μM of dNTPs, 330 ng of BSA, 0.14 μl of 5% (v/v) β-mercaptoethanol, 1 unit of Promega^® ^*Taq *polymerase, 20 pmol of forward primer, 20 pmol of reverse primer. The following cycling conditions were used: one incubation at 94°C for 5 min; 35 cycles of 94°C for 30 s; Tm for 30 s; 74°C for 1 min: followed by one incubation at 74°C for 5 min (where the Tm is appropriate for each set of primers).

### RT-PCR

To amplify the 5' end of *AnoACE7*, cDNA was prepared using an Invitrogen Cloned AMV kit as per the manufacturer's instructions. Using 2 μg total RNA from blood fed *A. gambiae *and 10 mM gene specific primer (ACE7 REV: 5'-GTCTATTCGGCGCATTTGAT-3'), first strand synthesis was performed at 50°C for 1 hour. PCR was then carried out using 1 μl of the first strand reaction, 2 mM dNTPs, 0.5 mM each primer (ACE7 REV and ACE7 FOR3: 5'-AGTGAAAACGTAACCATTGCAGAA-3') and 2.5 units Proofstart enzyme (Qiagen). PCR conditions were as follows: initial denaturation at 95°C, 5 min. then 35 cycles of 95°C for 30s, 54°C for 30s, 72°C for 3 min., and a final extension at 72°C for 5 min. A 1.1 kb product was purified using the Qiagen^® ^gel purification kit as per the manufacturer's instructions, cloned into pGEM^®^T-Easy and sequenced using M13 forward and reverse, and internal primers. The accession number of this sequence is [EMBL:AM085517]. For semi-quantitative RT-PCR, first strand synthesis of cDNA took place in a 20 μl reaction volume using 1 μg of RNA and Promega ImPromII RT^® ^kit as per the manufacturer's instructions and either random or gene-specific primers. PCR was carried out using the method described above with 2 μl of cDNA, primers as in Table 1, and the following cycling conditions: 94°C for 5 min. then 35 cycles of 94°C for 15 s, Tm for 15 s, 74°C for 30 s followed by incubation at 74°C for 5 min. PCR products were separated by agarose gel electrophoresis. To confirm identity of the PCR products, bands were purified using the Qiagen^® ^gel purification kit, cloned into pGEM^® ^T-Easy and sequenced using M13 forward and reverse primers.

### Quantitative RT-PCR

RNA (3 μg) was reverse transcribed using the Promega ImPromII^® ^kit and the resulting cDNA quantified using a Biorad iCycler instrument. cDNA (5 μl) was diluted in water to a total of 200 μl. Three-fold serial dilutions were made from this mixture as templates for the calibration curve. The remaining 15 μl of each experimental cDNA was made up to 400 μl with water. Reactions were performed in duplicate using 10 μl of template, 12.5 μl of ABsolute™ QPCR SYBR^® ^green and 2.5 μl of primers at a concentration determined to be optimum for each primer pair. Reactions were subjected to the following amplification cycles; 95°C for 15 min, once; 95°C for 15 s; 60°C for 30 s for 40 cycles. Melt curves were also performed to confirm that a single PCR product and no primer dimers were produced. The melt curves were performed using the following conditions; one incubation at 95°C for 30 s, 55°C for 30 s, once; followed by incubation at 55°C for 10 s 82 times, with a temperature increase of 0.5°C every cycle after cycle 2. Transcript levels of the AnoACE genes were normalised against the levels of mRNA for the ribosomal protein RPS17, amplified using primers 5'-TTGACCATGGATTTCGACAC-3' and 5'-TGATGGAAATACCACGCACT-3', forward and reverse, respectively.

## List of abbreviations

ACE: angiotensin I-converting enzyme, AnoACE: *Anopheles *ACE

## Authors' contributions

SB and AJL identified AnoACE genes and carried out expression studies. SB produced the initial draft of the manuscript. JAS cloned and sequenced the 5' end of AnoACE7. REI helped draft the manuscript. ADS refined the AnoACE annotation, carried out sequence comparisons and phylogenetic analysis, and re-drafted the manuscript. REI and ADS jointly planned the study and supervised experiments.

## Supplementary Material

Additional File 1 AnoACE protein sequences in FASTA format. Click here for file
